# Associations of overall and specific carbohydrate intake with anxiety status evolution in the prospective NutriNet-Santé population-based cohort

**DOI:** 10.1038/s41598-022-25337-5

**Published:** 2022-12-14

**Authors:** Junko Kose, Pauline Duquenne, Margaux Robert, Charlotte Debras, Pilar Galan, Sandrine Péneau, Serge Hercberg, Mathilde Touvier, Valentina A. Andreeva

**Affiliations:** 1 Nutritional Epidemiology Research Group (EREN), Sorbonne Paris Nord University, INSERM U1153, INRAE U1125, CNAM, Epidemiology and Statistics Research Center, University of Paris (CRESS), 74 Rue Marcel Cachin, 93017 Bobigny, France; 2 Department of Public Health, AP-HP Paris Seine-Saint-Denis Hospital System, Bobigny, France

**Keywords:** Anxiety, Risk factors

## Abstract

We investigated the association between carbohydrate intake and anxiety evolution within the general-population NutriNet-Santé cohort (N = 15,602; 73.8% female; mean age = 53.8y; mean follow-up = 5.4y). Carbohydrate intake was estimated at baseline from ≥ 2 24-h dietary records. Trait anxiety (STAI-T) was measured once at baseline (2013–2016) and once at follow-up (2020), resulting in 4 groups: “None” = absence of high anxiety (STAI-T > 40 points) at any time point; “Transient” = high anxiety only at baseline; “Onset at follow-up” = high anxiety only at follow-up; “Persistent” = high anxiety at baseline and follow-up. Polytomous logistic regression models revealed that sweetened beverage intake was associated with higher odds of “Transient” anxiety (OR_Q4vsQ1_ = 1.11; 95% CI 1.02–1.21). Intake of complex carbohydrates (OR_Q4vsQ1_ = 1.12; 1.01–1.25) was associated with higher odds of anxiety “Onset at follow-up.” The % energy from carbohydrates (OR_Q4vsQ1_ = 1.11; 1.03–1.19), intakes of total carbohydrates (OR_Q4vsQ1_ = 1.10; 1.03–1.18) and complex carbohydrates (OR_Q4vsQ1_ = 1.09; 1.02–1.17) were associated with higher odds of “Persistent” anxiety, whereas 100% fruit juice intake showed lower odds of “Persistent” anxiety (OR_Q4vsQ1_ = 0.87; 0.81–0.94). This prospective study found significant associations between dietary carbohydrate intake and anxiety status evolution among French adults. The findings could help inform dietary interventions aimed at anxiety prevention and management.

## Introduction

Anxiety disorders are characterized by excessive fear (emotional response to imminent threat) and apprehension (anticipation for future threat), typically lasting for 6 months or longer, and resulting in behavioral disturbance^1^. A systematic review of 48 studies from around the world reported pooled 1-year and lifetime prevalence estimates of 10.6% and 16.6% in adults, respectively^2^. The age-adjusted anxiety disorder prevalence is the highest among all mental disorders, with a substantial contribution to disability-adjusted life years^3^. Moreover, the COVID-19 pandemic has provoked an estimated global increase of 25.6% in anxiety disorder prevalence^4^, further highlighting the urgent need for primary and secondary prevention programs. One propitious intervention target pertains to dietary intake. For example, the deleterious impact of dietary sugar intake on depression has been well documented^5,6^, with plausible mechanisms related to gut microbiota dysbiosis, immune system dysregulation, oxidative stress, and inflammation^7,8^.

Regarding anxiety disorders, there is some, albeit inconsistent cross-sectional evidence of the link with overall and specific carbohydrate intake^9–11^, glycemic index/load^12^, insulin index^13^, soft drinks^14,15^, added sugars^16^, whole grains and fruit intake^17–24^. A scoping review of the relationship between diet and prevalence or severity of anxiety concluded that increased intake of sugar and refined carbohydrates was positively associated, while fruit intake was inversely associated with anxiety^25^. To our knowledge, there is no prospective epidemiological study on the link between carbohydrate intake and general anxiety conducted in a large population-based sample. However, some prospective and intervention studies with small or homogeneous samples exist. For example, a carbohydrate-focused intervention among 93 overweight/obese individuals reported non-significant effects on anxiety status^26^; likewise, a clinical intervention intended to increase fruit and vegetable intake among 171 young adults showed no effects of the intervention on anxiety^27^. A prospective study with pre-hypertensive or pre-diabetic Buddhist temple members reported a non-significant association between fruit intake and general anxiety^28^. Limitations of the existing epidemiological studies pertain to cross-sectional models preventing any inference of causality^9–24^, prospective studies using small or homogeneous samples (e.g., young adults)^26–29^, and modelling of non-specific mental health outcomes (e.g., general well-being)^30^. Furthermore, no prospective studies on the diet-anxiety link appear to have used Spielberger’s State-Trait Anxiety Inventory (STAI)—one of the most common anxiety measures^31^—in large heterogeneous samples^26,32,33^.

Therefore, the aim of the present study was to investigate the associations between overall and specific carbohydrate intake and anxiety status evolution using STAI in a large population-based prospective study. Our main hypothesis was that individuals with a higher refined carbohydrate intake would have an increased risk of anxiety and that those with a higher intake of foods known to have beneficial properties, such as fruit and whole grains, would have a lower risk of anxiety.

## Material and methods

### The NutriNet-Santé online cohort

NutriNet-Santé is a French, ongoing prospective cohort launched online in 2009. Details about its design, protocol, and main research goals were previously reported^34^. In brief, periodic multimedia-based recruitment calls target males and females aged 18 years and older who have the ability to follow an Internet-based study protocol (https://etude-nutrinet-sante.fr/).

At inclusion and once a year thereafter, participants are asked to fill out a set of five questionnaires focused on socio-demographic and lifestyle characteristics, anthropometrics, physical activity, diet (every 6 months), and health status. Additional nutrition- or health-related questionnaires are administered on a regular basis as part of the follow-up. All such questionnaires are completed on a voluntary basis and do not impact participant status.

**Figure 1:**
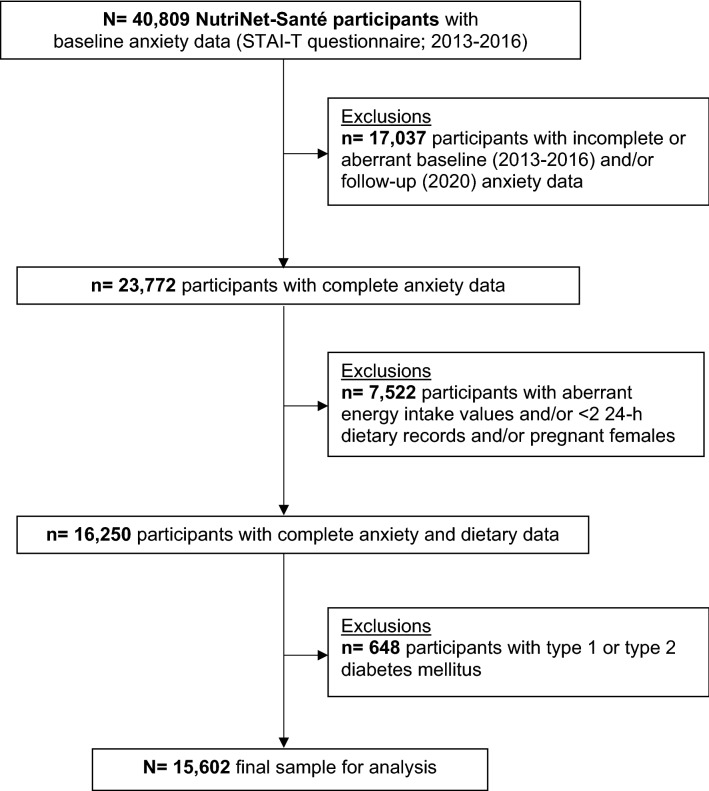
Participant selection flowchart.

For the present analysis, we selected individuals who had completed the anxiety assessment twice and who had at least 2 valid 24-h dietary records completed at baseline. Individuals with aberrant dietary data, with fewer than 2 dietary records, with prevalent or incident type 1 or type 2 diabetes, and those who were pregnant at the time of the dietary assessment were not eligible for the present analysis (Fig. 1).

### Measures

#### Dietary data

Carbohydrate intake was the main exposure in this analysis. In NutriNet-Santé, participants are asked to complete 24-h dietary records at baseline and every 6 months thereafter. Each assessment is intended to cover three non-consecutive days. The 24-h dietary record tool has been validated against both dietitian interviews and different nutritional status biomarkers^35–37^. Participants are asked to report each food, beverage, and composite dish consumed from midnight to midnight, including the portion size/quantity, estimated with the help of validated photographs^38^, the preparation method or recipe used, and each meal setting (place, time, etc.). Next, NutriNet-Santé’s own food composition table including > 3500 items is used to estimate mean daily calorie and nutrient intake^39^. All collected dietary data are weighted in order to respect the 5:7 and 2:7 ratios of weekdays and weekend days. For this analysis, each participant’s dietary carbohydrate intake was averaged across a minimum of two 24-h dietary records completed within a 2.5-year window around the baseline anxiety assessment date (described below); it was energy-adjusted using the residual method^40^.

The main exposure variables in the present study were the following carbohydrate intake measures: percentage of total calories from carbohydrates; total carbohydrates (*g/d*) (i.e., the sum of complex carbohydrates and simple sugars); complex carbohydrates (*g/d*) (starches and polyols); simple sugars (*g/d*) (i.e., sum of added sugars and sugar that is naturally-present in food such as fruit, dairy products and honey); added sugars (*g/d*); starch (*g/d*); fiber (*g/d*); whole grains (*g/d*); fresh fruit (*g/d*); fruit including dried fruit (*g/d*); sweet/sweetened foods (*g/d*); 100% fruit juice (*ml/d*); and sweet/sweetened beverages (*ml/d*).

Sweet/sweetened foods included milk-based desserts (creams, custards/puddings, milkshakes, sweetened yogurt and cottage cheese with sugar content > 12%), sweetened cereal products (breakfast cereal and cereal bars with sugar content ≥ 20%), cookies/cakes and pastries (all types of cookies, biscuits, cakes, baked goods, and pastries), confectionary (candies, sweets, honey, jam, table sugar, sweetened fruit purees, toppings, syrups, sweetened condensed milk, chocolate, ice cream, sorbets). Sweet/sweetened beverages included non-alcoholic sugar-sweetened beverages, fruit juices from concentrate, and nectars. Given the value distributions, participants were categorized into sex-specific quartiles for each exposure variable (Supplementary Table S1).

#### Anxiety assessment

Anxiety status was the main outcome in this analysis. It was assessed by self-reports using the validated French version of the trait anxiety subscale of STAI Form Y (STAI-T)^41^, once at baseline (2013–2016; n = 40,809) and once at follow-up (2020; n = 39,610). STAI is one of the most widely used tools to evaluate general anxiety proneness (as a state and as a trait), distinguishing it from depression^42^. Separate sets of 20 questions, scored on a 4-point Likert scale ranging from “Almost never” to “Almost always,” assess state and trait anxiety; each set has been the subject of a psychometric evaluation^42^. The higher the score, the greater the proneness to anxiety^42^. Considering the objectives of this study and for purposes of consistency with prior research^10,11^, we used only the trait-anxiety subscale (STAI-T). It assesses anxiety proneness as a relatively stable personality feature^31^. Trait anxiety measured by the STAI-T has been strongly correlated with generalized anxiety disorder^43^. As in prior research, and given the lack of an established cut-off, participants were considered as having “high general anxiety” if their total score was > 40 points or as having “low general anxiety” if their total score was ≤ 40 points^10^. Next, all participants were categorized in four groups according to their baseline and follow-up anxiety status: (1) “None” = absence of high general anxiety at any time point; (2) “Transient” = high general anxiety reported only at baseline; (3) “Onset at follow-up” = high general anxiety reported only at follow-up; (4) “Persistent” = high general anxiety reported at baseline and follow-up.

#### Covariate data

We used a validated socio-demographic questionnaire to collect self-reported data on age (*years*, continuous scale), sex, educational level (less than high school, high school diploma or equivalent, college/undergraduate degree, graduate degree), employment status (without professional activity including homemaker/disabled/unemployed/student, manual/blue collar worker, office work/administrative staff, professional/executive staff, retired), marital status (living alone or married/cohabiting), presence of children aged < 18y in the household (yes or no), alcohol use (*g* ethanol/*d*, continuous scale), and smoking status (never, former, current smoker)^44^; we also used a validated anthropometric questionnaire for self-reported height and weight^45,46^ which allowed the calculation of body mass index (BMI; kg/m^2^), thus classifying participants into four categories (underweight: < 18.5, normal weight: 18.5–24.9, overweight: 25.0–29.9, and obese: ≥ 30.0 kg/m^2^). The International Physical Activity Questionnaire—Short Form assessed sedentariness (minutes/d spent sitting) and physical activity levels based on an established scoring protocol (low, moderate, high)^47^. As all of the above questionnaires are administered at baseline and annually thereafter, we used covariate data collected within a 2.5-year window around the baseline STAI-T completion date.

Data on medication use for mental disorders (anxiety, addictive disorders, bipolar disorder, depression, anorexia nervosa, memory impairment, or sleep disorders) were obtained from the self-reported annual health status questionnaire. Depressive symptoms were assessed using the Center for Epidemiologic Studies Depression scale (CES-D)^48^ which is administrated every 2 years; thus, we used data closest to the baseline STAI-T date. The CES-D contains 20 items scored on a 4-point Likert scale ranging from “less than 1 day” to “5–7 days”, evaluating the frequency of experiencing depressive symptoms over the previous week. We used cut-offs validated for the French population (≥ 17 for males and ≥ 23 for females)^49^. Next, the likelihood of having an ED was screened in 2014 using the validated 5-item SCOFF questionnaire^50,51^. Each item is a Yes/No question, with two or more positive responses indicating a strong likelihood of ED.

### Statistical analysis

Descriptive and dietary characteristics across anxiety status reflect number (percent) from chi-squared tests (categorical variables) and mean (± SD) from ANOVA (continuous variables). We assessed the associations between sex-specific quartiles of carbohydrate intake (exposure) and anxiety status evolution from baseline to follow-up (outcome) using polytomous logistic regression (reference = lowest quartile of each nutrient/food intake; “None” for anxiety status) adjusted for age, sex, alcohol consumption, smoking status, physical activity level, sedentariness, educational level, employment status, marital status, presence of children aged < 18 years in the household, and number of 24-h dietary records. Tests for linear trend were performed by modeling a numeric value (0, 1, 2, 3) for each dietary quartile. We handled missing values for any covariates in the main model using the Multiple Imputation by Chained Equations method (20 imputed data sets)^52^. All tests were two-sided and *p* < 0.05 was considered as evidence for statistical significance. SAS version 9.4 (SAS Institute, Inc., Cary NC, USA) was used for all statistical analyses.

### Sensitivity analyses

Three sets of sensitivity analyses were conducted to test the robustness of the main results. In the first sensitivity analysis, an adjustment for self-reported prescribed medication use for mental disorders was added to the main model. In the second sensitivity analysis, an adjustment for presence of depressive symptoms and likelihood of eating disorders (ED) was added to the main model, because of potential associations not only between carbohydrate intake and depression^53^ and ED^54^, but also between these mental disorders^1^. Finally, in the third sensitivity analysis, we added all of these variables (i.e., medication use, presence of depressive symptoms, and likelihood of ED).

### Ethical standards

The NutriNet-Santé cohort study is conducted according to the Declaration of Helsinki guidelines. It was approved by the Institutional Review Board of the French Institute for Health and Medical Research (INSERM # 00000388FWA00005831) and by the National Commission on Informatics and Liberty (CNIL # 908450 and # 909216). NutriNet-Santé is registered at: https://clinicaltrials.gov/ct2/show/NCT03335644. Electronic informed consent was obtained from all volunteers prior to inclusion in the cohort.

## Results

### Description of sample

In the final sample (N = 15,602), 73.8% of the participants were female and the mean age was 53.8 ± 13.1 years. Participants included in the analysis were generally older, more likely to be male, retired, and never smokers, to report higher levels of both physical activity and educational attainment, and were less likely to be obese, current smokers, or to live alone compared to those excluded from the analysis (all *p* < 0.0001; data not tabulated). The distribution of participants according to anxiety status was as follows: “None” n = 8939 (57.3%); “Transient” n = 1956 (12.5%); “Onset at follow-up” n = 1200 (7.7%); “Persistent” n = 3507 (22.5%). Mean follow-up time in the study was 5.4 ± 0.9 years. Table 1 presents the participants’ characteristics across anxiety status evolution. In general, individuals with high anxiety at least once during the study period were more likely to be female, younger, underweight or obese, current smokers, sedentary, without a professional activity, to live alone or to have children in the household, to have lower levels of physical activity, to consume less alcohol, and were less likely to be retired compared to those in the group “None” (all *p* < 0.0001). Table 2 presents participants’ baseline dietary data according to anxiety category. In the full sample, the mean number of 24-h dietary records was 8.7 ± 3.8.

**Table 1:** Baseline descriptive characteristics of the participants according to anxiety status^a^ (N = 15,602; NutriNet-Santé cohort; France).

	None		Transient anxiety		Onset of anxiety at follow-up		Persistent anxiety		*p*-value^b^
	n = 8939		n = 1956		n = 1200		n = 3507		
**T-STAI score at baseline**	31.4	(5.3)	45.9	(5.0)	35.8	(3.9)	50.3	(7.0)	< 0.0001
**T-STAI score at follow-up**	29.9	(5.5)	35.0	(4.3)	45.9	(5.2)	49.8	(6.9)	< 0.0001
**Sex**									
Female	6047	(67.7)	1543	(78.9)	964	(80.3)	2962	(84.5)	< 0.0001
Male	2892	(32.4)	413	(21.1)	236	(19.7)	545	(15.5)	
**Age, years, mean (SD)**	55.5	(12.7)	52.1	(12.9)	51.0	(14.4)	51.1	(13.3)	< 0.0001
**Age category**									
18–39 y	1262	(14.1)	399	(20.4)	318	(26.5)	813	(23.2)	< 0.0001
40–59 y	3597	(40.2)	923	(47.2)	474	(39.5)	1555	(44.3)	
≥ 60 y	4080	(45.6)	634	(32.4)	408	(34.0)	1139	(32.5)	
**Educational level**^**c**^									
Less than high school	1158	(13.8)	243	(13.2)	134	(11.8)	432	(13.2)	0.01
High school diploma or equivalent	1484	(17.7)	307	(16.7)	169	(14.9)	571	(17.4)	
College, undergraduate degree	2423	(28.9)	551	(29.9)	388	(34.2)	1018	(31.0)	
Graduate degree	3335	(39.7)	743	(40.3)	444	(39.1)	1260	(38.4)	
**Employment status**^**d**^									
Without professional activity (homemaker, disabled, unemployed, student)	524	(6.0)	156	(8.3)	100	(8.5)	373	(11.0)	< 0.0001
Manual/blue collar	996	(11.4)	300	(15.9)	184	(15.7)	604	(17.8)	
Office work/administrative staff	1310	(15.0)	332	(17.6)	223	(19.0)	590	(17.4)	
Professional/executive staff	2070	(23.6)	536	(28.3)	275	(23.4)	785	(23.1)	
Retired	3865	(44.1)	568	(30.0)	394	(33.5)	1046	(30.8)	
**Marital status**^**e**^									
Living alone (single, divorced, widowed)	1851	(20.9)	535	(27.8)	254	(21.3)	952	(27.5)	< 0.0001
Married/cohabiting	6999	(79.1)	1391	(72.2)	937	(78.7)	2513	(72.5)	
**Children aged < 18 y in household**^**f**^									
No	6931	(77.9)	1441	(74.2)	859	(71.8)	2537	(72.8)	< 0.0001
Yes	1969	(22.1)	501	(25.8)	337	(28.2)	949	(27.2)	
**Physical activity level**^**g,h**^									
Low	1633	(18.6)	476	(24.8)	279	(23.7)	899	(26.1)	< 0.0001
Moderate	3539	(40.2)	768	(40.0)	497	(42.2)	1467	(42.6)	
High	3630	(41.2)	676	(35.2)	402	(34.1)	1076	(31.3)	
**Sedentariness (minutes spent sitting/d), mean (SD)**^**g,i**^	342.5	(183.7)	370.5	(195.0)	358.0	(190.2)	371.6	(225.4)	< 0.0001
**Body Mass Index (BMI, kg/m**^**2**^**), mean (SD)**	23.7	(3.7)	23.6	(4.1)	23.7	(3.9)	23.4	(4.4)	< 0.01
**BMI category**									
Underweight (< 18.5)	316	(3.5)	106	(5.4)	55	(4.6)	240	(6.8)	< 0.0001
Normal weight (18.5–24.9)	5975	(66.8)	1255	(64.2)	772	(64.3)	2284	(65.1)	
Overweight (25.0–29.9)	2148	(24.0)	463	(23.7)	295	(24.6)	719	(20.5)	
Obese (≥ 30.0)	500	(5.6)	132	(6.8)	78	(6.5)	264	(7.5)	
**Smoking status**^**j**^									
Never smoker	4494	(50.8)	1032	(53.6)	606	(51.0)	1865	(53.9)	< 0.0001
Former smoker	3598	(40.7)	716	(37.2)	455	(38.3)	1243	(35.9)	
Current smoker	753	(8.5)	178	(9.2)	128	(10.8)	354	(10.2)	
**Alcohol use, g ethanol/d, mean (SD)**^**k**^	8.3	(10.5)	7.0	(9.7)	6.8	(9.3)	6.4	(8.9)	< 0.0001

Values refer to number (%) except when noted otherwise. Values are rounded off to one decimal place.

^a^Anxiety status assessed with the trait subscale of Spielberger’s State-Trait Anxiety Inventory form Y (STAI-T). STAI -T score > 40 points considered as high general anxiety; anxiety categories as follows: (1) None = no reported high general anxiety at any time point; (2) Transient anxiety = high general anxiety reported only at baseline; (3) Onset of anxiety at follow-up = high general anxiety reported only at follow-up; (4) Persistent anxiety = high general anxiety reported at baseline and follow-up.

^b^*P*-values obtained from chi-squared tests or ANOVA, as appropriate.

^c^“Educational level” contained 942 missing values that were imputed prior to the main analysis.

^d^“Employment status” contained 371 missing values that were imputed prior to the main analysis.

^e^“Marital status” contained 170 missing values that were imputed prior to the main analysis.

^f^“Children aged < 18 y in household” contained 78 missing values that were imputed prior to the main analysis.

^g^Assessed with the International Physical Activity Questionnaire-Short Form according to established scoring criteria.

^h^“Physical activity” contained 260 missing values that were imputed prior to the main analysis.

^i^“Sedentariness” contained 275 missing values that were imputed prior to the main analysis.

^j^“Smoking status” contained 180 missing values that were imputed prior to the main analysis.

^k^“Alcohol use” contained 167 missing values that were imputed prior to the main analysis.

**Table 2:** Baseline dietary data according to anxiety status^a^ (N = 15,602; NutriNet-Santé cohort; France).

	None		Transient anxiety		Onset of anxiety at follow-up		Persistent anxiety		*p*-value^b^
	n = 8939		n = 1956		n = 1200		n = 3507		
Total energy intake (Kcal/d)	1954.1	(449.5)	1897.2	(422.9)	1889.7	(426.3)	1873.1	(415.1)	< 0.0001
Number of 24-h dietary records	9.0	(3.7)	8.4	(3.7)	8.4	(3.8)	8.4	(3.7)	< 0.0001
% of energy from carbohydrates	41.2	(6.2)	41.3	(5.9)	41.5	(5.9)	41.7	(6.5)	< 0.01
% of energy from protein	16.3	(2.7)	16.2	(2.8)	16.3	(2.9)	16.2	(2.9)	0.29
% of energy from fat	39.0	(5.4)	39.4	(5.1)	39.1	(5.2)	39.3	(5.8)	< 0.01
Total carbohydrates (g/d)	201.1	(39.8)	195.7	(35.4)	196.3	(35.1)	194.4	(35.4)	< 0.0001
Complex carbohydrates (g/d)	105.8	(29.7)	102.4	(26.8)	103.5	(27.1)	102.1	(27.0)	< 0.0001
Simple sugars (g/d)	94.8	(25.1)	92.8	(23.1)	92.1	(22.5)	91.8	(24.6)	< 0.0001
Added sugars (g/d)	37.6	(17.4)	38.1	(17.2)	36.9	(16.3)	37.9	(18.0)	0.22
Starch (g/d)	105.8	(29.8)	102.4	(26.8)	103.6	(27.3)	102.0	(26.9)	< 0.0001
Fiber (g/d)	21.0	(6.1)	20.0	(5.8)	20.2	(6.3)	20.0	(6.2)	< 0.0001
Whole grains (g/d)	39.5	(46.1)	34.5	(39.8)	36.5	(43.1)	35.4	(42.6)	< 0.0001
Fresh fruit (g/d)	210.6	(137.7)	196.2	(131.0)	195.5	(138.0)	194.0	(141.6)	< 0.0001
Fruit including dried fruit (g/d)	213.7	(139.0)	199.0	(132.3)	198.4	(139.5)	196.8	(142.6)	< 0.0001
Sweet/sweetened foods except for fruit (g/d)	146.3	(71.4)	147.9	(68.9)	146.6	(66.8)	149.2	(72.1)	0.20
100% fruit juice (ml/d)	48.4	(68.6)	49.6	(69.2)	48.1	(69.4)	43.4	(62.9)	< 0.001
Sweet/sweetened beverages except for 100% fruit juice (ml/d)	21.9	(53.4)	28.5	(69.5)	24.0	(57.3)	26.4	(69.9)	< 0.0001

Nutrient/food intakes were adjusted for total energy intake by the residual method. Values refer to mean (SD). Values are rounded off to one decimal place, except the *p*-values. 1 kcal = 4.1868 kJ.

^a^Anxiety status assessed with the trait subscale of Spielberger’s State-Trait Anxiety Inventory form Y (STAI-T). STAI-T score > 40 points considered as high general anxiety; anxiety categories as follows: (1) None = no reported high general anxiety at any time point; (2) Transient anxiety = high general anxiety reported only at baseline; (3) Onset of anxiety at follow-up = high general anxiety reported only at follow-up; (4) Persistent anxiety = high general anxiety reported at baseline and follow-up.

^b^*P*-values obtained from ANOVA.

### Association between overall and specific carbohydrate intake and anxiety status evolution

Table 3 shows the main results obtained with adjusted polytomous logistic regression. Sweet/sweetened beverage intake was associated with higher odds of “Transient” anxiety (OR_Q4vsQ1_ = 1.11 (95% Confidence Interval (CI): 1.02–1.21), *p*_trend_ < 0.02). Complex carbohydrate intake and starch intake showed increased odds regarding anxiety “Onset at follow-up” (OR_Q4vsQ1_ = 1.12 (95% CI 1.01–1.25), *p*_trend_ < 0.02 and OR_Q4vsQ1_ = 1.13 (95% CI 1.02–1.25), *p*_trend_ < 0.01, respectively). For “Persistent” anxiety, the percentage of energy from carbohydrates (OR_Q4vsQ1_ = 1.11 (95% CI 1.03–1.19), *p*_trend_ < 0.02), intakes of total carbohydrates (OR_Q4vsQ1_ = 1.10 (95% CI 1.03–1.18), *p*_trend_ < 0.02), complex carbohydrates (OR_Q4vsQ1_ = 1.09 (95% CI 1.02–1.17), *p*_trend_ < 0.01), and starch (OR_Q4vsQ1_ = 1.09 (95% CI 1.01–1.16), *p*_trend_ < 0.02) were associated with increased odds. In contrast, 100% fruit juice intake showed lower odds of “Persistent” anxiety (OR_Q4vsQ1_ = 0.87 (95% CI 0.81–0.94), *p*_trend_ < 0.01). No other significant results were observed.

**Table 3:** Associations between quartiles of carbohydrate intake and anxiety status^a^ (N = 15,602; NutriNet-Santé cohort; France).

	Transient anxiety				Onset of anxiety at follow-up				Persistent anxiety			
	n = 1956				n = 1200				n = 3507			
	Quartile	OR^b^	95% CI^b^	*p* for trend	Quartile	OR^b^	95% CI^b^	*p* for trend	Quartile	OR^b^	95% CI^b^	*p* for trend
% of energy from carbohydrates	2	1.05	(0.97–1.15)	0.92	2	1.08	(0.97–1.19)	0.11	2	0.99	(0.93–1.07)	** < 0.02**
	3	1.05	(0.97–1.14)		3	1.00	(0.90–1.11)		3	0.97	(0.90–1.04)	
	4	0.95	(0.87–1.04)		4	1.07	(0.96–1.19)		**4**	**1.11**	**(1.03–1.19)**	
Total carbohydrates (g/d)	2	1.06	(0.97–1.15)	0.74	2	1.05	(0.95–1.16)	0.06	2	0.99	(0.92–1.06)	** < 0.02**
	3	1.07	(0.98–1.16)		3	0.99	(0.89–1.10)		3	0.98	(0.92–1.06)	
	4	0.93	(0.85–1.01)		4	1.09	(0.98–1.21)		**4**	**1.10**	**(1.03–1.18)**	
Complex carbohydrates (g/d)	2	1.08	(0.99–1.18)	0.65	2	0.99	(0.89–1.10)	** < 0.02**	2	0.96	(0.90–1.03)	** < 0.01**
	3	0.99	(0.91–1.08)		3	1.01	(0.91–1.12)		3	1.01	(0.94–1.08)	
	4	1.00	(0.91–1.09)		**4**	**1.12**	**(1.01–1.25)**		**4**	**1.09**	**(1.02–1.17)**	
Simple sugars (g/d)	2	0.97	(0.89–1.06)	0.71	2	1.20	(1.08–1.32)	0.49	2	0.95	(0.88–1.02)	0.57
	3	1.10	(1.01–1.20)		3	0.95	(0.86–1.06)		3	1.01	(0.94–1.08)	
	4	0.96	(0.88–1.05)		4	0.95	(0.85–1.05)		4	0.99	(0.92–1.07)	
Added sugars (g/d)	2	1.01	(0.92–1.10)	0.06	2	1.09	(0.98–1.20)	0.42	2	0.97	(0.91–1.04)	0.18
	3	1.08	(0.99–1.17)		3	0.96	(0.87–1.07)		3	0.97	(0.90–1.04)	
	4	1.02	(0.94–1.12)		4	0.96	(0.86–1.07)		4	1.08	(1.00–1.15)	
Starch (g/d)	2	1.06	(0.98–1.16)	0.66	2	0.98	(0.88–1.09)	** < 0.01**	2	0.97	(0.90–1.04)	** < 0.02**
	3	1.01	(0.93–1.10)		3	1.01	(0.91–1.13)		3	1.01	(0.94–1.08)	
	4	0.99	(0.91–1.08)		**4**	**1.13**	**(1.02–1.25)**		**4**	**1.09**	**(1.01–1.16)**	
Fiber (g/d)	2	1.04	(0.96–1.13)	0.16	2	1.07	(0.96–1.18)	0.80	2	0.97	(0.90–1.04)	0.76
	3	0.95	(0.87–1.04)		3	1.02	(0.91–1.13)		3	0.93	(0.86–0.99)	
	4	0.96	(0.88–1.05)		4	0.98	(0.88–1.10)		4	1.05	(0.98–1.13)	
Whole grains (g/d)	2	1.00	(0.92–1.09)	0.10	2	0.99	(0.89–1.10)	0.79	2	1.01	(0.95–1.09)	** < 0.05**
	3	1.08	(0.99–1.17)		3	1.06	(0.95–1.18)		3	0.99	(0.92–1.06)	
	4	0.89	(0.81–0.97)		4	0.95	(0.86–1.06)		4	0.94	(0.88–1.01)	
Fresh fruit (g/d)	2	0.96	(0.88–1.04)	0.61	2	1.10	(1.00–1.22)	0.59	2	0.94	(0.88–1.01)	0.06
	3	1.13	(1.04–1.23)		3	0.93	(0.83–1.04)		3	1.01	(0.94–1.08)	
	4	0.92	(0.84–1.00)		4	0.99	(0.89–1.11)		4	0.95	(0.89–1.03)	
Fruit including dried fruit (g/d)	2	0.95	(0.87–1.04)	0.79	2	1.09	(0.98–1.21)	0.75	2	0.95	(0.89–1.02)	0.13
	3	1.13	(1.04–1.23)		3	0.95	(0.85–1.06)		3	0.99	(0.92–1.06)	
	4	0.92	(0.84–1.01)		4	0.99	(0.89–1.11)		4	0.97	(0.91–1.05)	
Sweet/sweetened food except fruit (g/d)	2	1.00	(0.92–1.09)	0.45	2	1.03	(0.93–1.14)	0.67	2	0.93	(0.87–1.00)	0.09
	3	1.08	(0.99–1.18)		3	1.01	(0.91–1.12)		3	1.03	(0.96–1.10)	
	4	0.98	(0.90–1.07)		4	1.00	(0.90–1.12)		4	1.06	(0.99–1.13)	
100% fruit juice (ml/d)	2	0.96	(0.88–1.04)	0.83	2	1.01	(0.90–1.12)	0.72	2	1.03	(0.96–1.11)	** < 0.01**
	3	1.03	(0.95–1.12)		3	1.10	(0.99–1.22)		3	1.05	(0.98–1.13)	
	4	1.00	(0.92–1.09)		4	0.92	(0.83–1.02)		**4**	**0.87**	**(0.81–0.94)**	
Sweet/sweetened beverages except 100% fruit juice (ml/d)	2	0.91	(0.83–0.99)	** < 0.02**	2	1.03	(0.93–1.14)	0.79	2	0.90	(0.84–0.97)	0.68
	3	1.02	(0.94–1.11)		3	1.06	(0.95–1.18)		3	1.03	(0.96–1.10)	
	**4**	**1.11**	**(1.02–1.21)**		4	0.94	(0.84–1.05)		4	1.03	(0.96–1.11)	

^a^Anxiety status assessed with the trait subscale of Spielberger’s State-Trait Anxiety Inventory form Y (STAI-T). STAI-T score > 40 points considered as high general anxiety; anxiety categories as follows: (1) None = no reported high general anxiety at any time point; (2) Transient anxiety = high general anxiety reported only at baseline; (3) Onset of anxiety at follow-up = high general anxiety reported only at follow-up; (4) Persistent anxiety = high general anxiety reported at baseline and follow-up.

^b^Results from multivariable polytomous logistic regression (reference categories: anxiety status = None (n = 8939) and lowest quartile of carbohydrate intake) adjusted for age (time-scale), BMI (continuous variable), sex, number of 24-h dietary records, smoking status, educational level, employment status, physical activity level, sedentariness, alcohol intake, marital status, and presence of children aged < 18 y in household.

Values are rounded off to two decimal places. Significant results are shown in bold.

### Sensitivity analyses

Results of the three sets of sensitivity analyses are presented in Supplementary Tables S2, S3, and S4. Overall, similar results to those obtained in the main analyses were observed. One difference pertained to 100% fruit juice whose association with anxiety became non-significant in the model with an additional adjustment for depressive symptoms and likelihood of ED.

## Discussion

This large, prospective epidemiological study revealed some significant relationships between carbohydrate intake and anxiety status evolution (i.e., “None,” “Transient,” “Onset at follow-up” and “Persistent”). Our hypotheses that participants with a higher refined carbohydrate intake would have an increased risk of anxiety and that those with a higher intake of fruit and whole grains would have a reduced risk of anxiety were only partially supported. Increased odds of “Persistent” anxiety were observed among participants with higher intakes of total and complex carbohydrates, and starch. None of carbohydrate measure was associated with all three anxiety categories. Also, contrary to our hypothesis, we found no significant associations of intake of fiber, fruit, whole grains or sweet/sweetened food with any of the anxiety categories in our study. That may be due to a true absence of an association. It should be noted, however, that the sweet/sweetened food variable was heterogeneous in nature, including a variety of items, which might have diluted the associations with anxiety. Also, the generally low participation rate of people with mental disorders in epidemiological studies has been discussed^55^; these factors might have led to an underestimation of the associations that might partly explain the null findings in the present study.

In turn, we observed that the consumption of sweet/sweetened beverages was linked to higher odds of “Transient” anxiety, which was defined as high general anxiety only at baseline. Given no significant results for “Onset at follow-up” or for “Persistent” anxiety, the observed association might be of short duration or it might be an indication of reverse causality. Prior cross-sectional studies have also reported positive associations between intake of soft drinks and anxiety status^14,15^. Soft drinks are regarded as “comfort food” whose intake might be subject to affect-related triggers, including anxiety^56^. Next, in our study there was a borderline association between added sugar intake and “Transient” anxiety, while previous cross-sectional studies have reported a positive association^10,16^. Our results may be driven by the fact that added sugars included various types (e.g., glucose, fructose, etc.,), which may have differential relationships with anxiety. Indeed, a previous study on the link between intake of various types of simple sugars and depression reported a significant inverse association only for lactose^57^. More research with detailed data on sugar type is needed to elucidate the findings. Next, in our study, intakes of complex carbohydrates and starch, but not whole grains, were associated with high general anxiety only at follow-up. To our knowledge, no prospective research on the relationship between starch intake and anxiety risk has been conducted. However, one large study on the incidence of depression, which is often comorbid with anxiety disorders^1^, reported null findings as regards starch intake, a significant protective association with progressively increasing whole grains intake, and a significant detrimental association with increasing refined grains intake^57^. A cross-sectional study with young and middle-aged adults reported a positive association between refined grain intake and anxiety^20^. Also, a scoping review concluded that refined carbohydrate intake was linked to an increased prevalence or severity of anxiety^25^. Increased consumption of refined grains—high in starch and low in fiber—can cause hyperglycemia, whose effect on the inflammatory response has been evoked^58^. Pro-inflammatory cytokines have an effect on neuroinflammation which is well known as a major etiological factor of mental disorders in the diet-mental health domain^8^. Next, the percentage of energy from carbohydrates, intakes of total and complex carbohydrates, and starch were significantly associated with “Persistent” anxiety in our analysis. The significant results for percentage of energy from carbohydrates and total carbohydrates might be partly driven by complex carbohydrate and starch intake, given the absence of significant results regarding simple or added sugars in the main analysis. Next, lower odds only for “Persistent” anxiety were observed with 100% fruit juice consumption, suggesting chronicity of the behavior. 100% fruit juice is rich in monosaccharides, but also in antioxidants^59^ which have known protective properties against mental disorders^8^. In the sensitivity analysis with an additional adjustment for presence of depressive symptoms and likelihood of ED, however, the results for 100% fruit juice were no longer significant. Thus, our results merit confirmation before firm conclusions could be drawn.

Limitations of the present study must be recognized. First, the main outcome was general anxiety assessed using a self-reported validated questionnaire (which does not provide data on anxiety onset), reflecting a relatively stable personality feature that is not easily subject to change over time^31^. Even though general anxiety estimated with the STAI-T has been strongly correlated with generalized anxiety disorder^43^, it does not correspond to a clinical diagnosis. It should be noted, however, that anxiety disorders are likely under-diagnosed in the general population^60^. Using an Internet-based platform could minimize social desirability and encourage participants to provide personal information^61^, arguing for the use of self-reported anxiety measures in population-based epidemiological research. In addition, even though continuous measures are generally more sensitive than are categorical ones, we dichotomized the anxiety variable at baseline and at follow-up in order to facilitate interpretation of the results regarding anxiety status evolution. Second, despite the inclusion of a number of covariates in the main and supplementary statistical analyses, the existence of non-measured residual confounding cannot be excluded. Third, participants with prevalent or incident diabetes mellitus (type 1 or type 2) were excluded from the analyses, because of specific carbohydrate intake regimens. Next, the follow-up anxiety assessment (March–September 2020) coincided with the beginning of the COVID-19 pandemic which disrupted the way of life and the well-being of the general population worldwide. Our study, however, was focused on trait rather than state anxiety, thus it is believed that the outcome measure was only minimally impacted by the context. In addition, the relatively long follow-up time (5.4 ± 0.9 years) is seen as an important strength of the study, allowing the interpretation of prospective associations. Finally, caution is advised when generalizing the findings, owing to the response rate and the fact that individuals who do not provide follow-up mental disorder information might be more likely to suffer from mental disorders than those who do provide such data^62,63^, underscoring the possibility of under-estimation of the observed associations. Also, there are differences between NutriNet-Santé participants and the French general population (i.e., a higher proportion of females, individuals with higher educational and socio-economic levels in the cohort)^64^.

Despite these limitations, to our knowledge, this is the first large-scale epidemiological study to reveal prospective associations between carbohydrate intake from various sources and anxiety status in a heterogenous sample of adults. The data were collected with validated questionnaires^41,44–46^; moreover, carbohydrate intake was estimated from a mean of nearly nine 24-h dietary records, previously validated against dietitian interviews and against various biomarkers of nutritional status^35–37^. Considering the heavy and growing burden of anxiety disorders^2,3^, the results of our study as well as future research in this domain could contribute to public health needs assessment and to the development of dietary interventions aimed at anxiety prevention and management. Future prospective studies could strengthen the evidence and confirm the present findings.

## Supplementary Information

Supplementary Tables.

## Data Availability

Researchers at public institutions can submit a project collaboration request that includes information about their institution and a brief description of the project to: collaboration@etude-nutrinet-sante.fr. All requests are reviewed by the steering committee of the NutriNet-Santé study. In case of approval, a signed data access agreement will be requested and additional authorizations from the competent administrative authorities may be needed regarding human subjects’ data protection. In accordance with existing regulations, no personally identifiable data will be made available.

## Abbreviations

BMI: Body Mass Index
CES-D: Center for Epidemiologic Studies Depression Scale
CI: Confidence interval
ED: Eating disorders
OR: Odds ratio
Q: Quartile
STAI-T: Trait subscale of Spielberger’s State-Trait Anxiety Inventory
