# Supplementary material for: Associations of overall and specific carbohydrate intake with anxiety status evolution in the prospective NutriNet-Santé population-based cohort
Source: Sci Rep. 2022 Dec 14;12:21647. doi: 10.1038/s41598-022-25337-5 (PMC9750050; doi:10.1038/s41598-022-25337-5)
Supplement: Supplementary file 1 — Supplementary Tables. [file 41598_2022_25337_MOESM1_ESM.pdf]

Supplementary Table S1. Values of the sex-specific quartiles of each nutrient/food intake[1] used in the main analysis (N= 15,602; NutriNet-Santé cohort; France)

		1st quartile	Median	3rd quartile
**% energy from carbohydrates**	Male	37.2	41.4	45.4
	Female	37.5	41.3	45.1
**Total carbohydrates (g/d)**	Male	213.7	237.4	259.5
	Female	168.5	184.8	201.6
**Complex carbohydrates (g/d)**	Male	110.5	128.6	147.4
	Female	81.3	94.1	108.4
**Simple sugars (g/d)**	Male	88.7	105.3	123.3
	Female	75.4	88.0	101.6
**Added sugars (g/d)**	Male	30.4	42.0	54.7
	Female	25.0	34.3	44.5
**Starch (g/d)**	Male	110.1	128.4	147.4
	Female	81.5	94.3	108.7
**Fiber (g/d)**	Male	18.9	22.4	27.0
	Female	15.8	18.9	22.5
**Whole grains (g/d)**	Male	4.1	23.5	66.1
	Female	5.7	23.9	54.3
**Fresh fruit (g/d)**	Male	116.3	200.2	308.1
	Female	106.6	118.0	263.1
**Fruit including dried fruits (g/d)**	Male	118.8	202.4	311.9
	Female	108.9	180.8	266.1
**Sweet/sweetened food except fruit (g/d)**	Male	106.5	151.2	206.5
	Female	96.2	136.1	181.3
**100% fruit juice (ml/d)**	Male	3.0	18.8	99.3

	Female	2.6	14.8	70.4
**Sweet/sweetened beverages except 100% fruit juice (ml/d)**	Male	-0.6	8.0	25.2
	Female	-0.1	5.4	21.6

[1] Nutrient/food intakes were adjusted for total energy intake by the residual method.

Supplementary Table S2. Sensitivity analyses of the association between quartiles of mean carbohydrate intake and anxiety status[1] with an additional adjustment for self-reported medication use for mental disorders (N= 15,602; NutriNet-Santé cohort; France)

	Transient anxiety n=1,956				Onset of anxiety at follow-up n=1,200				Persistent anxiety n=3,507			
	Quartile	OR[2]	95% CI[2]	*p* for trend	Quartile	OR[2]	95% CI[2]	*p* for trend	Quartile	OR[2]	95% CI[2]	*p* for trend
% of energy from carbohydrates	2	1.05	(0.96-1.14)	1.00	2	1.08	(0.97-1.19)	0.10	2	0.99	(0.92-1.06)	**<0.01**
	3	1.05	(0.97-1.15)		3	1.00	(0.90-1.11)		3	0.97	(0.90-1.05)	
	4	0.95	(0.87-1.04)		4	1.07	(0.96-1.19)		**4**	**1.11**	**(1.04-1.20)**	
Total carbohydrates (g/d)	2	1.06	(0.98-1.16)	0.82	2	1.05	(0.95-1.17)	0.05	2	0.99	(0.92-1.06)	**<0.01**
	3	1.07	(0.99-1.17)		3	0.99	(0.89-1.10)		3	0.99	(0.92-1.07)	
	4	0.93	(0.85-1.01)		4	1.09	(0.98-1.22)		**4**	**1.10**	**(1.02-1.18)**	
Complex carbohydrates (g/d)	2	1.08	(0.99-1.17)	0.49	2	0.99	(0.89-1.10)	**<0.01**	2	0.95	(0.89-1.02)	**<0.01**
	3	1.00	(0.92-1.09)		3	1.01	(0.91-1.12)		3	1.02	(0.95-1.10)	
	4	1.00	(0.92-1.09)		**4**	**1.13**	**(1.02-1.25)**		**4**	**1.11**	**(1.03-1.19)**	
Simple sugars (g/d)	2	0.97	(0.89-1.06)	0.82	2	1.20	(1.08-1.32)	0.42	2	0.95	(0.88-1.02)	0.31
	3	1.09	(1.00-1.19)		3	0.95	(0.85-1.06)		3	0.99	(0.92-1.07)	
	4	0.96	(0.88-1.05)		4	0.94	(0.85-1.05)		4	0.99	(0.92-1.06)	

Added sugars (g/d)	2	1.00	(0.92-1.09)	0.09	2	1.08	(0.98-1.20)	0.37	2	0.97	(0.90-1.04)	0.36
	3	1.07	(0.99-1.17)		3	0.96	(0.86-1.07)		3	0.96	(0.89-1.03)	
	4	1.02	(0.93-1.11)		4	0.96	(0.86-1.07)		4	1.07	(1.00-1.15)	
Starch (g/d)	2	1.06	(0.97-1.15)	0.50	2	0.98	(0.88-1.09)	**<0.01**	2	0.96	(0.89-1.03)	**<0.01**
	3	1.02	(0.93-1.11)		3	1.02	(0.92-1.13)		3	1.02	(0.95-1.09)	
	4	1.00	(0.91-1.09)		**4**	**1.14**	**(1.02-1.26)**		**4**	**1.10**	**(1.03-1.18)**	
Fiber (g/d)	2	1.04	(0.95-1.13)	0.16	2	1.06	(0.96-1.18)	0.81	2	0.96	(0.89-1.03)	0.72
	3	0.96	(0.88-1.04)		3	1.02	(0.92-1.13)		3	0.94	(0.87-1.01)	
	4	0.96	(0.88-1.05)		4	0.98	(0.87-1.10)		4	1.05	(0.97-1.13)	
Whole grains (g/d)	2	1.00	(0.92-1.09)	0.06	2	0.99	(0.89-1.10)	0.68	2	1.02	(0.95-1.09)	**<0.02**
	3	1.07	(0.98-1.16)		3	1.05	(0.94-1.16)		3	0.96	(0.90-1.03)	
	4	0.89	(0.81-0.97)		4	0.95	(0.86-1.06)		4	0.95	(0.88-1.02)	
Fresh fruit (g/d)	2	0.96	(0.88-1.05)	0.70	2	1.11	(1.00-1.23)	0.64	2	0.96	(0.89-1.03)	0.11
	3	1.13	(1.04-1.23)		3	0.93	(0.83-1.04)		3	1.01	(0.94-1.08)	
	4	0.92	(0.84-1.01)		4	0.99	(0.89-1.11)		4	0.96	(0.89-1.03)	
Fruit including dried	2	0.95	(0.87-1.04)	0.88	2	1.09	(0.99-1.21)	0.80	2	0.96	(0.89-1.03)	0.20
fruit (g/d)	3	1.14	(1.04-1.24)		3	0.95	(0.85-1.06)		3	0.99	(0.92-1.07)	
	4	0.92	(0.84-1.01)		4	0.99	(0.89-1.11)		4	0.98	(0.91-1.05)	

Sweet/sweetened food except fruit (g/d)	2	1.00	(0.92-1.09)	0.93	2	1.03	(0.93-1.15)	0.99	2	0.93	(0.87-1.00)	0.63
	3	1.08	(0.99-1.17)		3	1.01	(0.91-1.12)		3	1.02	(0.95-1.09)	
	4	0.97	(0.89-1.06)		4	1.00	(0.90-1.11)		4	1.05	(0.98-1.13)	
100% fruit juice (ml/d)	2	0.96	(0.88-1.05)	0.81	2	1.01	(0.91-1.12)	0.72	2	1.04	(0.97-1.12)	**<0.01**
	3	1.02	(0.94-1.11)		3	1.09	(0.99-1.21)		3	1.03	(0.96-1.11)	
	4	1.01	(0.93-1.10)		4	0.92	(0.83-1.03)		**4**	**0.88**	**(0.82-0.95)**	
Sweet/sweetened beverages except 100% fruit juice (ml/d)	2	0.91	(0.83-0.99)	**<0.03**	2	1.03	(0.93-1.14)	0.70	2	0.90	(0.84-0.97)	0.93
	3	1.02	(0.94-1.11)		3	1.06	(0.95-1.17)		3	1.02	(0.95-1.10)	
	**4**	**1.11**	**(1.02-1.21)**		4	0.94	(0.84-1.05)		4	1.03	(0.96-1.11)	

[1] Anxiety status assessed with the trait subscale of Spielberger's State-Trait Anxiety Inventory form Y (STAI-T). STAI -T score >40 points considered as high general anxiety; anxiety categories as follows: 1) None= no reported high general anxiety at any time point; 2) Transient anxiety= high general anxiety reported only at baseline; 3) Onset of anxiety at follow-up= high general anxiety reported only at follow-up; 4) Persistent anxiety= high general anxiety reported at baseline and follow-up.

[2] Results from multivariable polytomous logistic regression (reference categories: anxiety status=None (n=8,939) and lowest quartile of carbohydrate intake) adjusted for age (time-scale), BMI (continuous variable), sex, number of 24-hour dietary records, smoking status, educational level, employment status, physical activity level, sedentariness, alcohol intake, marital status, presence of children aged <18 y in household, and self-reported

medication use for mental disorders (anxiety, addictive disorders, bipolar disorders, depression, anorexia nervosa, memory impairment, or sleep disorders).

Values are rounded off to two decimal places. Significant results are shown in bold.

Supplementary Table S3. Sensitivity analyses of the association between quartiles of mean carbohydrate intake and anxiety status[1] with an additional adjustment for presence of depressive symptoms and likelihood of eating disorders (N= 15,329; NutriNet-Santé cohort; France)[2]

	Transient anxiety n=1,923				Onset of anxiety at follow-up n=1,176				Persistent anxiety n=3,433			
	Quartile	OR[3]	95% CI[3]	*p* for trend	Quartile	OR[3]	95% CI[3]	*p* for trend	Quartile	OR[3]	95% CI[3]	*p* for trend
% of energy from carbohydrates	2	1.06	(0.97-1.16)	0.93	2	1.10	(0.99-1.22)	0.15	2	1.00	(0.93-1.08)	**<0.01**
	3	1.06	(0.97-1.16)		3	1.01	(0.91-1.13)		3	1.00	(0.93-1.08)	
	4	0.95	(0.86-1.04)		4	1.04	(0.93-1.16)		**4**	**1.11**	**(1.02-1.20)**	
Total carbohydrates (g/d)	2	1.06	(0.98-1.16)	0.87	2	1.07	(0.96-1.19)	0.09	2	0.99	(0.91-1.06)	**<0.01**
	3	1.08	(0.99-1.18)		3	1.01	(0.90-1.12)		3	1.01	(0.94-1.09)	
	4	0.92	(0.84-1.01)		4	1.06	(0.95-1.19)		**4**	**1.10**	**(1.02-1.19)**	
Complex carbohydrates (g/d)	2	1.08	(0.99-1.18)	0.45	2	1.00	(0.90-1.11)	**<0.01**	2	0.96	(0.89-1.04)	**<0.01**
	3	1.00	(0.92-1.09)		3	1.01	(0.91-1.13)		3	1.04	(0.97-1.12)	
	4	1.00	(0.92-1.09)		**4**	**1.12**	**(1.01-1.24)**		**4**	**1.11**	**(1.03-1.19)**	
Simple sugars (g/d)	2	0.97	(0.89-1.06)	0.80	2	1.21	(1.09-1.33)	0.36	2	0.94	(0.87-1.02)	0.37
	3	1.10	(1.01-1.20)		3	0.96	(0.86-1.07)		3	1.01	(0.94-1.09)	
	4	0.96	(0.88-1.05)		4	0.93	(0.83-1.04)		4	0.98	(0.91-1.06)	

Added sugars (g/d)	2	1.00	(0.92-1.09)	0.07	2	1.10	(0.99-1.22)	0.43	2	0.97	(0.90-1.04)	0.36
	3	1.10	(1.01-1.20)		3	0.98	(0.88-1.09)		3	1.00	(0.93-1.08)	
	4	1.01	(0.92-1.11)		4	0.95	(0.85-1.06)		4	1.04	(0.97-1.13)	
Starch (g/d)	2	1.07	(0.98-1.16)	0.46	2	0.98	(0.88-1.09)	**<0.01**	2	0.98	(0.90-1.05)	**<0.01**
	3	1.02	(0.94-1.12)		3	1.03	(0.92-1.14)		3	1.04	(0.96-1.12)	
	4	0.99	(0.91-1.09)		**4**	**1.12**	**(1.01-1.25)**		**4**	**1.10**	**(1.02-1.18)**	
Fiber (g/d)	2	1.05	(0.97-1.15)	0.11	2	1.09	(0.98-1.21)	0.98	2	0.99	(0.92-1.07)	0.75
	3	0.95	(0.87-1.03)		3	1.00	(0.90-1.12)		3	0.94	(0.87-1.02)	
	4	0.95	(0.87-1.05)		4	0.97	(0.86-1.08)		4	1.03	(0.95-1.12)	
Whole grains (g/d)	2	1.00	(0.91-1.09)	0.08	2	0.98	(0.88-1.09)	0.89	2	0.99	(0.91-1.06)	0.06
	3	1.07	(0.98-1.16)		3	1.05	(0.94-1.16)		3	0.96	(0.89-1.04)	
	4	0.89	(0.81-0.98)		4	0.97	(0.87-1.08)		4	0.97	(0.9-1.05)	
Fresh fruit (g/d)	2	0.97	(0.89-1.06)	0.38	2	1.13	(1.01-1.25)	0.46	2	0.97	(0.89-1.04)	**<0.05**
	3	1.13	(1.04-1.24)		3	0.92	(0.83-1.03)		3	1.04	(0.96-1.12)	
	4	0.89	(0.81-0.98)		4	0.98	(0.87-1.09)		4	0.92	(0.85-1.00)	
Fruit including dried	2	0.96	(0.88-1.05)	0.50	2	1.11	(1.00-1.23)	0.59	2	0.97	(0.89-1.04)	0.08
fruit (g/d)	3	1.15	(1.06-1.25)		3	0.95	(0.85-1.06)		3	1.02	(0.95-1.10)	
	4	0.90	(0.82-0.98)		4	0.97	(0.87-1.09)		4	0.94	(0.87-1.01)	

Sweet/sweetened food except fruit (g/d)	2	1.01	(0.93-1.11)	0.48	2	1.04	(0.94-1.16)	0.58	2	0.95	(0.88-1.02)	0.23
	3	1.08	(0.99-1.18)		3	1.01	(0.91-1.13)		3	1.02	(0.95-1.10)	
	4	0.97	(0.89-1.06)		4	1.01	(0.90-1.12)		4	1.05	(0.97-1.13)	
100% fruit juice (ml/d)	2	0.94	(0.86-1.03)	0.61	2	0.99	(0.88-1.10)	0.86	2	0.99	(0.92-1.07)	0.11
	3	1.02	(0.94-1.12)		3	1.10	(0.99-1.22)		3	1.05	(0.98-1.14)	
	4	1.03	(0.94-1.12)		4	0.94	(0.84-1.04)		4	0.92	(0.85-0.99)	
Sweet/sweetened beverages except 100% fruit juice (ml/d)	2	0.91	(0.83-0.99)	**<0.05**	2	1.03	(0.93-1.15)	0.40	2	0.91	(0.85-0.99)	0.34
	3	1.02	(0.93-1.11)		3	1.05	(0.94-1.17)		3	1.00	(0.93-1.08)	
	**4**	**1.10**	**(1.01-1.20)**		4	0.92	(0.82-1.03)		4	1.00	(0.93-1.08)	

[1] Anxiety status assessed with the trait subscale of Spielberger's State-Trait Anxiety Inventory form Y (STAI-T). STAI -T score >40 points considered as high general anxiety; anxiety categories as follows: 1) None= no reported high general anxiety at any time point; 2) Transient anxiety= high general anxiety reported only at baseline; 3) Onset of anxiety at follow-up= high general anxiety reported only at follow-up; 4) Persistent anxiety= high general anxiety reported at baseline and follow-up.

[2] n= 28 individuals in the main sample did not have data for depressive symptoms; n=249 individuals in the main sample did not have data for eating disorder likelihood, and 273 individuals in the main sample did not have data for both of these variables.

[3] Results from multivariable polytomous logistic regression (reference categories: anxiety status=None (n=8,797) and lowest quartile of carbohydrate intake) adjusted for age (time-scale), BMI (continuous variable), sex, number of 24-hour dietary records, smoking status, educational level,

employment status, physical activity level, sedentariness, alcohol intake, marital status, presence of children aged <18 y in household, presence of depressive symptoms (CES-D score ≥17 for males and ≥23 for females), and likelihood of eating disorders (SCOFF questionnaire score ≥2 positive responses).

Values are rounded off to two decimal places. Significant results are shown in bold.

Supplementary Table S4. Sensitivity analyses of the association between quartiles of mean carbohydrate intake and anxiety status[1] with an additional adjustment for medication use for mental disorders, presence of depressive symptoms and likelihood of eating disorders (N= 15,329; NutriNet-Santé cohort; France)[2]

	Transient anxiety n=1,923				Onset of anxiety at follow-up n=1,176				Persistent anxiety n=3,433			
	Quartile	OR[3]	95% CI[3]	*p* for trend	Quartile	OR[3]	95% CI[3]	*p* for trend	Quartile	OR[3]	95% CI[3]	*p* for trend
% of energy from carbohydrates	2	1.06	(0.97-1.15)	0.86	2	1.10	(0.99-1.22)	0.13	2	1.00	(0.92-1.08)	**<0.01**
	3	1.07	(0.98-1.16)		3	1.02	(0.91-1.13)		3	1.00	(0.93-1.09)	
	4	0.95	(0.86-1.04)		4	1.04	(0.93-1.16)		**4**	**1.11**	**(1.03-1.20)**	
Total carbohydrates (g/d)	2	1.06	(0.98-1.16)	0.94	2	1.07	(0.96-1.19)	0.08	2	0.99	(0.91-1.07)	**<0.01**
	3	1.09	(1.00-1.18)		3	1.01	(0.91-1.12)		3	1.02	(0.94-1.10)	
	4	0.92	(0.84-1.01)		4	1.07	(0.95-1.19)		**4**	**1.10**	**(1.02-1.19)**	
Complex carbohydrates (g/d)	2	1.08	(0.99-1.17)	0.37	2	0.99	(0.89-1.11)	**<0.01**	2	0.96	(0.89-1.04)	**<0.01**
	3	1.01	(0.92-1.10)		3	1.02	(0.92-1.13)		3	1.05	(0.97-1.13)	
	4	1.01	(0.92-1.10)		**4**	**1.12**	**(1.01-1.25)**		**4**	**1.11**	**(1.03-1.20)**	
Simple sugars (g/d)	2	0.97	(0.89-1.06)	0.86	2	1.21	(1.09-1.34)	0.34	2	0.95	(0.88-1.02)	0.25
	3	1.09	(1.00-1.19)		3	0.95	(0.86-1.07)		3	1.00	(0.92-1.08)	

	4	0.96	(0.87-1.05)		4	0.93	(0.83-1.04)		4	0.98	(0.90-1.06)	
Added sugars (g/d)	2	1.00	(0.92-1.09)	0.09	2	1.10	(0.99-1.22)	0.39	2	0.97	(0.89-1.04)	0.51
	3	1.09	(1.00-1.19)		3	0.97	(0.87-1.08)		3	0.99	(0.91-1.07)	
	4	1.01	(0.92-1.11)		4	0.95	(0.85-1.06)		4	1.04	(0.96-1.13)	
Starch (g/d)	2	1.06	(0.97-1.16)	0.38	2	0.98	(0.88-1.09)	**<0.01**	2	0.97	(0.9-1.05)	**<0.01**
	3	1.03	(0.94-1.12)		3	1.03	(0.93-1.15)		3	1.04	(0.97-1.13)	
	4	1.00	(0.91-1.09)		**4**	**1.13**	**(1.01-1.25)**		**4**	**1.11**	**(1.03-1.2)**	
Fiber (g/d)	2	1.05	(0.96-1.14)	0.11	2	1.09	(0.98-1.21)	0.96	2	0.98	(0.91-1.06)	0.76
	3	0.95	(0.87-1.04)		3	1.00	(0.90-1.12)		3	0.95	(0.88-1.03)	
	4	0.95	(0.87-1.04)		4	0.97	(0.86-1.08)		4	1.03	(0.95-1.11)	
Whole grains (g/d)	2	1.00	(0.91-1.09)	0.06	2	0.98	(0.88-1.09)	0.79	2	0.99	(0.92-1.07)	**<0.04**
	3	1.06	(0.97-1.15)		3	1.04	(0.93-1.15)		3	0.94	(0.87-1.02)	
	4	0.89	(0.82-0.98)		4	0.97	(0.87-1.08)		4	0.97	(0.90-1.05)	
Fresh fruit (g/d)	2	0.98	(0.89-1.07)	0.47	2	1.13	(1.02-1.25)	0.52	2	0.97	(0.90-1.05)	0.09
	3	1.14	(1.04-1.24)		3	0.93	(0.83-1.04)		3	1.03	(0.96-1.12)	
	4	0.90	(0.82-0.98)		4	0.98	(0.88-1.10)		4	0.93	(0.86-1.01)	
Fruit including dried	2	0.96	(0.88-1.05)	0.59	2	1.11	(1.00-1.23)	0.65	2	0.97	(0.90-1.05)	0.14
fruit (g/d)	3	1.15	(1.06-1.26)		3	0.95	(0.85-1.07)		3	1.02	(0.95-1.11)	

	4	0.90	(0.82-0.99)		4	0.98	(0.87-1.09)		4	0.94	(0.87-1.02)	
Sweet/sweetened	2	1.02	(0.93-1.11)	0.57	2	1.04	(0.94-1.16)	0.65	2	0.95	(0.88-1.03)	0.36
food except fruit	3	1.07	(0.98-1.17)		3	1.01	(0.90-1.12)		3	1.01	(0.94-1.09)	
(g/d)	4	0.97	(0.89-1.06)		4	1.00	(0.90-1.12)		4	1.04	(0.96-1.12)	
100% fruit juice	2	0.94	(0.86-1.03)	0.58	2	0.99	(0.89-1.10)	0.88	2	1.00	(0.92-1.08)	0.11
(ml/d)	3	1.01	(0.93-1.11)		3	1.09	(0.98-1.21)		3	1.03	(0.96-1.12)	
	4	1.03	(0.95-1.13)		4	0.94	(0.84-1.05)		4	0.93	(0.86-1.00)	
Sweet/sweetened	2	0.91	(0.83-0.99)	**<0.05**	2	1.03	(0.93-1.15)	0.39	2	0.91	(0.84-0.99)	0.33
beverages except	3	1.02	(0.93-1.11)		3	1.05	(0.94-1.17)		3	1.00	(0.92-1.08)	
100% fruit juice	**4**	**1.10**	**(1.01-1.20)**		4	0.92	(0.82-1.03)		4	1.00	(0.92-1.08)	
(ml/d)												

[1] Anxiety status assessed with the trait subscale of Spielberger's State-Trait Anxiety Inventory form Y (STAI-T). STAI -T score >40 points considered as high general anxiety; anxiety categories as follows: 1) None= no reported high general anxiety at any time point; 2) Transient anxiety= high general anxiety reported only at baseline; 3) Onset of anxiety at follow-up= high general anxiety reported only at follow-up; 4) Persistent anxiety= high general anxiety reported at baseline and follow-up.

[2] n= 28 individuals in the main sample did not have data for depressive symptoms; n=249 individuals in the main sample did not have data for eating disorder likelihood, and 273 individuals in the main sample did not have data for both of these variables.

[3] Results from multivariable polytomous logistic regression (reference categories: anxiety status=None (n=8,797) and lowest quartile of carbohydrate intake) adjusted for age (time-scale), BMI (continuous variable), sex, number of 24-hour dietary records, smoking status, educational level, employment status, physical activity level, sedentariness, alcohol intake, marital status, presence of children aged <18 y in household, medication use for mental disorders (anxiety, addictive disorders, bipolar disorders, depression, anorexia nervosa, memory impairment, or sleep disorders), presence of depressive symptoms (CES-D score ≥17 for males and ≥23 for females), and likelihood of eating disorders (SCOFF questionnaire score ≥2 positive responses).

Values are rounded off to two decimal places. Significant results are shown in bold.
